# T Cell Subpopulations in the Physiopathology of Fibromyalgia: Evidence and Perspectives

**DOI:** 10.3390/ijms21041186

**Published:** 2020-02-11

**Authors:** Giuseppe Banfi, Marco Diani, Paolo D. Pigatto, Eva Reali

**Affiliations:** 1 IRCCS Istituto Ortopedico Galeazzi, 20161Milan, Italy; banfi.giuseppe@hsr.it (G.B.); marco.diani@unimi.it (M.D.); paolo.pigatto@unimi.it (P.D.P.); 2 School of Medicine, Università Vita-Salute San Raffaele, 20132 Milan, Italy; 3 Department of Biomedical, Surgical and Dental Sciences, University of Milan, 20122 Milan, Italy

**Keywords:** environmental sensitivity illnesses, immune responses, neuroimmunology, inflammation, pain, musculoskeletal diseases

## Abstract

Fibromyalgia is one of the most important “rheumatic” disorders, after osteoarthritis. The etiology of the disease is still not clear. At the moment, the most defined pathological mechanism is the alteration of central pain pathways, and emotional conditions can trigger or worsen symptoms. Increasing evidence supports the role of mast cells in maintaining pain conditions such as musculoskeletal pain and central sensitization. Importantly, mast cells can mediate microglia activation through the production of proinflammatory cytokines such as IL-1β, IL-6, and TNFα. In addition, levels of chemokines and proinflammatory cytokines are enhanced in serum and could contribute to inflammation at systemic level. Despite the well-characterized relationship between the nervous system and inflammation, the mechanism that links the different pathological features of fibromyalgia, including stress-related manifestations, central sensitization, and dysregulation of the innate and adaptive immune responses is largely unknown. This review aims to provide an overview of the current understanding of the role of adaptive immune cells, in particular T cells, in the physiopathology of fibromyalgia. It also aims at linking the latest advances emerging from basic science to envisage new perspectives to explain the role of T cells in interconnecting the psychological, neurological, and inflammatory symptoms of fibromyalgia.

## 1. Background

Fibromyalgia often comes to the attention of the orthopedic clinics because of the widespread chronic pain and musculoskeletal symptoms such as back and neck pain. The prevalence varies from 2% to 8% of the population [1]. The female:male ratio is 2:1 and can develop at any age, including in childhood. The prevalence is similar all over the world and industrialized countries do not have a higher prevalence. In a clinical setting, fibromyalgia is suspected in patients with multifocal pain not completely explained by inflammation or injury. In most cases, pain pathways throughout the body are amplified and musculoskeletal pain represents an important feature., The disease is also characterized by the presence of comorbidities, in particular chronic headaches, visceral pain, and sensory hyper-responsiveness, as well as psychiatric conditions such as depression and anxiety. Recently, it has been reported that in fibromyalgia patients, the presence of comorbid visceral pain such as irritable bowel syndrome is associated with increased severity of fibromyalgia symptoms [2].

The etiology of the disease is still not clear. Initially, fibromyalgia was defined as a chronic pain syndrome occurring in highly stressed individuals. Now it is known that there is not a single trigger [3,4,5] and the most defined pathological mechanism is the alteration of central pain pathways or central sensitization, associated with the amplification of the perception of pain even in the absence of the noxious stimuli. Moreover, emotional conditions can trigger or worsen symptoms [6,7,8,9,10]. Despite the well-known connection between the nervous system and inflammation, the mechanism that links the different pathological features of fibromyalgia, including stress-related manifestations, anxiety, central sensitization, and dysregulation of the inflammatory responses, is largely unknown.

In the early 1950s, the concept of neuroimmunology emerged that explored the crosstalk between immune cells and the nervous system. Over the decades, the role of the innate immunity in the onset of pain and stress-related manifestations has been partly characterized, whereas the role of the adaptive immune system is still poorly defined [11,12].

In fibromyalgia, the hypothalamic–pituitary–adrenal axis (HPA) is considered to play a role in the establishment of central sensitization [13,14]. 

It is known that the HPA axis controls response to stress and its activation leads to the secretion of corticotropin-releasing hormone (CRH), which modulates the immune response through the secretion of glucocorticoids. Patients with centralized pain syndromes can display hyper- or hypocortisolism and altered downstream signaling from the HPA axis. This includes increased infiltration of mast cells that also express receptors for CRH [13]. Release of granule from activated mast cells can lead to sensitization of peripheral and central nociceptors and the increase of pro-inflammatory cytokines [8,9,10,15]. 

In patients with fibromyalgia, the levels of corticotropin-releasing hormone (CRH), were increased in the serum and cerebrospinal fluid and correlated with the severity of pain [4,5,16,17]. Accordingly, the stress response has been reported to worsen fibromyalgia syndrome and to augment the pain responses [4,5]. Moreover, animal models of cold, restraint, and sound stress can manifest behavioral and inflammatory dysregulation associated with hyperalgesia, similar to what is observed in fibromyalgia patients [18,19,20]. 

In addition to the regulation of stress and inflammatory response, the HPA axis and the sympathetic–adrenal–medullary axis are also involved in the modulation of adaptive immunity with different effects. 

This review aims to provide an overview of the current understanding of the role of the cells of adaptive immunity, in particular T cells, in the physiopathology of fibromyalgia. It also aims at linking the latest advances emerging from basic science to envisage new perspectives that could explain the role of T cells in interconnecting the neurological and inflammatory symptoms of fibromyalgia. 

## 2. Inflammatory Mediators and Innate Immunity in the Generation of Pain in Fibromyalgia

Recent research proposed that fibromyalgia could involve localized inflammation in the hypothalamus [6,7]. This perspective arises from the evidence that increased levels of proinflammatory chemokines such as IL-8 were found in the serum and cerebrospinal fluid of patients with fibromyalgia [21,22,23,24]. Chemokines, in this case, could directly assist nociception through the binding to chemokine receptors present along the pain pathway [25,26]. In addition to IL-8, the cytokines IL-1β, TNFα, IL-6, and IL-17 were also enhanced in serum and could contribute to the inflammatory response in the central nervous system [27]. In plasma, the increased IL-17 levels correlated with the levels of TNFα, and IL-17 in cerebrospinal fluid and serum was found to correlate with pain and anxiety [28,29,30]. In addition, the serum level of IL-6 in patients was correlated with the pain score and, in animal models, the injection of IL-6, IL-1β, and TNFα was shown to induce behavioral changes [15]. 

Pain in fibromyalgia involves neuroinflammatory processes triggered by mast cells and microglia [31,32,33,34,35]. Mounting evidence indeed supports the role of mast cells in fibromyalgia comorbid disorders and painful conditions [6,36,37]. For example, CCL11 (eotaxin) and CCL2 (a potent chemoattractant for mast cells), were found to be elevated in the plasma of fibromyalgia patients [38]. In rat models, CCL2 induced widespread chronic muscle pain through the activation of its receptor, CCR2, on the peripheral nerve terminals [39]. On the other hand, in vitro, myoblasts treated with CCL2 were shown to secrete significant amounts of the key pro-inflammatory cytokine IL-1β as well as CXCL10 [38]

The role of mast cells in establishing pain has also been shown in a mouse model of postoperative pain, in which mast cell depletion prevented mechanical central pain sensitization [40].

In the interplay with mast cells, microglia which are the resident macrophages of the central nervous system, can have a role by increasing inflammation through the secretion of cytokines [41,42].

It has been proposed that CX3CL1 chemokine could represent the link between the signaling pathway of neuropathic pain and the immune responses. Soluble CX3CL1 is shed from primary afferent terminals and spinal neurons through the action of cathepsin S, which is released by activated microglial cells. CX3CL1 could, in turn, stimulate microglia thus establishing positive feedback. Therefore it is possible that chemokines and cytokines secreted by activated microglia can be involved in establishing a state of neuronal hyperactivity and central sensitization [13,43,44,45,46]. Overall, microglia, through these mechanisms, can contribute to brain inflammation and to the pathogenesis of different brain disorders [41,47,48,49,50,51,52]. Together these studies underline the role of cells and mediators of the innate immunity in maintaining pain conditions such as musculoskeletal pain and central sensitization [53].

It is, however, important to note that a very novel finding in the transgenic experimental mouse model showed that nociceptor stimulation of peripheral sensory afferent neurons was able to induce IL-17 secretion by local TCRγδ T cells and by CD4^+^ T cells [54]. Despite the increasing evidence in this field, the interconnections between the main symptoms of fibromyalgia, such as stress, anxiety, chronic musculoskeletal pain, and the adaptive immune responses still need an explanation.

## 3. Role of T Cells in Fibromyalgia: Literature Data

As compared to myalgic encephalomyelitis, in which the adaptive immune system has been described as a player in the pathogenesis, the association of fibromyalgia with infections and with the generation of cross-reactive antibodies is less clear and autoreactive immune responses are likely to play a less determinant role [55]. Nevertheless, changes in specific subsets of T cells have been reported in the literature even if the results are not conclusive.

The immune system can modulate the nervous system and vice versa. For example, the neurotransmitters, such as glutamate can trigger T cell functions and dopamine, gonadotrophin-releasing hormones, somatostatin, and substance P, were shown to affect the phenotypic polarization of T cells [56]. Animal studies have enlightened the role of T cells in pain development. In particular, the depletion of CD4^+^T cells in a mouse model was able to inhibit thermal hyperalgesia and tactile central sensitization following partial sciatic nerve ligation [57,58].

Mice lacking T cells and mice lacking IFNγ, which is mainly expressed by T cells, showed significantly less mechanical hypersensitivity. This suggests that T-cells are involved in neuropathic pain and that IFNγ plays a major role.

Furthermore, it has been shown that following injury, CD3, CD4, and CD8 T-cell markers were increased in female mice, more than in males. [59]. This latter finding underlines that T cells can be involved in pain development and should also be taken into account in the explanation of the female prevalence of fibromyalgia.

To clarify the potential role of T cells in fibromyalgia, we searched the PubMed database between 1960 and 2019 using search terms fibromyalgia and T cells (or lymphocytes). We found 28 articles. Only articles in English were selected; articles not directly addressing the issue of the involvement of T cells in fibromyalgia were excluded from the data extraction.

The first study on this topic referred to the altered interleukin-2 (IL-2) secretion in patients with primary fibromyalgia syndrome. IL-2 was chosen as an indicator of CD4^+^ response as it is secreted by T cells upon T cell receptor (TcR) stimulation and mediates T cell proliferation. The study was performed on isolated CD4^+^ T cells from 12 patients and control subjects. CD4^+^ T cells from patients required a higher concentration of the mitogen concanavalin A in order to reach optimal secretion of IL-2, thus indicating a lower response of CD4^+^ T cells from fibromyalgia patients to activating stimuli [60].

A subsequent study was performed on 65 patients with fibromyalgia, and lymphocyte subpopulations were analyzed, including CD3^+^T cells, CD19 (B cells), CD16 (natural killer cells), CD4^+^ T helper, and CD8^+^ cytotoxic T cells. The expression of the activation markers CD25, CD69, and CD71 on T cells was also evaluated. The results indicated that the number of CD3^+^T cells expressing the activation markers CD69 and CD25 was decreased in patients with fibromyalgia, whereas the CD4/CD8 ratio was similar in patients and controls. [61]

It followed a clinical study designed to analyze the immunophenotype and killing activity of peripheral blood mononuclear cell subpopulations in patients at baseline and upon treatment with low-doses of human IFNα. At baseline, 124 patients with fibromyalgia showed a higher percentage of T lymphocytes expressing the activation marker CD25 than healthy controls subjects. After IFNα treatment, the main difference observed was a decrease in the percentage of CD4^+^ T cells with activated phenotype (HLA-DR^+^CD4^+^) in the circulation. [62].

Another cohort of patients diagnosed with fibromyalgia according to American College of Rheumatology criteria was studied for cell-mediated sensitivity to environmental chemicals. Memory T lymphocytes were analyzed for proliferation in vitro in response to different chemical substances. In patients with fibromyalgia, the authors observed a significantly higher T cell proliferation in response to aluminum, lead, and platinum; and to a low extent to cadmium and silicon [63]. This evidence is also supported by results from other studies, indicating that people who have a history of fibromyalgia showed increased levels of metals (e.g., mercury, cadmium, cobalt, iron), leading to the release of pro-inflammatory cytokines. In particular, exposure to mercury could be associated with fibromyalgia/myalgic encephalomyelitis in humans [64,65,66,67].

A different group showed that victims of post-traumatic stress disorders have higher circulating T lymphocytes and lower cortisol levels, thus suggesting a risk for the development of autoimmune diseases. Patients in this study showed increased T-cell counts, hyperreactive immune responses on standardized delayed cutaneous hypersensitivity tests, and higher immunoglobulin-M levels [68]. In line with the association with autoimmune diseases, there is also considerable evidence indicating that autoimmune thyroid disease is associated with fibromyalgia [69].

Importantly, a study by Kaufmann and coworkers examined the effect of pain and stress on lymphocyte numbers, the lymphocyte subpopulations, and the Th1/Th2 cytokine ratio in T cells. Analysis of lymphocyte subpopulations indicated a significant reduction in the numbers of cytotoxic CD8^+^ lymphocytes in chronic pain patients whereas the ratio CD4/CD8 was not significantly altered [3]. Even if the results were not conclusive, the study enlightens the importance of the variations in the distribution of T cell subpopulations as a consequence of chronic stress. A negative correlation was indeed evidenced between post-traumatic stress scores and the number of CD8^+^ T cells in patients with fibromyalgia.

In an attempt to define whether also genetic predisposing factors influencing the response to serotonin may associate with T cell dysregulation, the polymorphic promoter region of the serotonin transporter (5-HTTLPR) was analyzed in patients with fibromyalgia. Patients were subdivided on the basis of the presence of the two allelic forms of 5-HTTLPR (either in homozygosis or in heterozygosis) and were evaluated for salivary cortisol levels and the absolute number of leucocyte subsets, such as natural killer (NK) cells and activated T and B lymphocytes. All patients had reduced cortisol levels. The homozygote group for the long allelic variant had increased CD4^+^CD25^low^ activated T lymphocytes, and homozygotes for the short allelic variant displayed elevated CD4^+^ HLA-DR^+^ activated T lymphocytes [8]. This suggests that there are no clear differences in the activation state of CD4^+^ T cells in patients expressing the different allelic variants, but there is a general increase in CD4^+^ T cells expressing intermediate or late activation markers in patients with fibromyalgia.

A more recent investigation on lymphocyte subsets in fibromyalgia analyzed Natural Killer T (NKT)-like CD3^+^CD56^+^ cells as possible mediators in mental symptoms such as depression in 96 fibromyalgia patients with anxiety and depression. Analysis of the frequency in the different subgroups of depression in fibromyalgia patients showed significant differences and the use of antidepressants significantly altered the level of CD3^+^CD56^+^NKT cells [9].

Patients with fibromyalgia also had increased levels of the inflammatory chemokines CCL17, CXCL9, CCL22, and CXCL11, in addition to CCL11 [70]. Taken together, the higher level of cytokines and chemokines exerting their chemotactic activity on cells of both innate and adaptive immunity suggests that the establishment of fibromyalgia syndrome and systemic inflammation could involve both cells of the innate immunity and T cells [70,71].

Analysis of the percentage of mucosal-associated invariant T (MAIT) cells in peripheral blood and cell surface marker expression in fibromyalgia patients showed a decrease in the MAIT cell population as compared to healthy control subjects. Among the cell surface antigens in MAIT cells, CCR7, the chemokine receptor which mediates the homing to the lymph nodes, and the CD27 T cell costimulatory molecule were significantly increased. By contrast, there was a decrease in the expression of the chemokine receptors, CCR4 and CXCR1, and of the NK receptor, NKp80. These results suggested that MAIT cells may have a phenotype that could result in an increase in the response and production of proinflammatory cytokines.

In these patients, the drug treatment interruption resulted in alteration of the expression of CCR4, CCR5, CXCR4, CD27, CD28, ICOS CD127 (IL-7 receptor alpha), CD94, NKp80, and the activation marker CD69 in MAIT cells [3,72].

A comprehensive study on the T cell compartment has been recently published by Guggino et al., focusing the attention on the role of CD4^+^ T cells subpopulations in the pathophysiology of fibromyalgia. In a cohort of 36 patients, analysis of the circulating CD4^+^ T cell subsets showed an increased Th1 type signature in fibromyalgia patients compared to controls. In CD4^+^ T cells stimulated with PMA-Ionomycin, there was a significant increase in IFNγ and TNFα production. Interestingly, in these patients, serum levels of TNF-α and IFN-γ were also increased and correlated with disease severity.

Together, these results indicate the expansion of Th1 cell subpopulations and a general trend toward higher levels of inflammatory cytokine-producing CD4^+^ cells in subjects with fibromyalgia compared with those with unrelated diseases. These patients underwent hyperbaric oxygen therapy, which has been studied for analgesic effect in a model of nociceptive neuroinflammatory pain and has been used for the treatment of several pain conditions. Interestingly a significant reduction of circulating cells with Th1 phenotype was observed after therapy that was correlated with an improvement of the disease score. In these patients, serotonin levels in the serum were reduced and showed an inverse correlation with the disease severity [73].

Recently, research has emerged focusing on cannabinoids as immune-modulating agents with an effect on T-cells, B-cells, monocytes, and microglia. As cannabinoids can cause a general reduction in pro-inflammatory cytokine levels and an enhancement in anti-inflammatory cytokines, a putative role in the treatment of multiple sclerosis, inflammatory bowel disease, and fibromyalgia has been suggested [74,75].

Together, the literature data on the role of T cells in fibromyalgia are not conclusive (Table 1). Regarding the activation state, it is important to mention that the protocol used for surface marker detection, the cohort of patients, and the phase of the disease, as well as the pharmacological treatment, may affect the results as the expression of activation markers can be different in acute versus chronic phases. Overall the concept that mainly emerges from the analysis of the results of these studies is an increase in the function and percentage of CD4^+^ T cells with a Th1 signature in fibromyalgia patients.

It is unclear however how the changes in T lymphocyte subset distribution and in the activation state can be linked to stress and pain conditions and other fibromyalgia manifestations. At present, it is also unclear whether subgroups of patients with different comorbid diseases can display differences in the T cell subset distribution. Except for the work of Nugraha et al. analyzing the role of NKT-like cells in the development of psychiatric comorbidities and an earlier report, there are no clear indications in this direction [9,76]. It would be of interest to expand the knowledge in this field as it has been shown that comorbid diseases such as visceral pain could amplify fibromyalgia symptoms [2].

## 4. T Cell Mediated Immune Response in Fibromyalgia: New Perspectives

An interesting perspective about the role of adaptive immunity in disease physiopathology has been provided by Shoenfeld and coworkers in one article of this collection. The authors proposed the hypothesis of an autoimmune nature of the disease. This hypothesis is based on the evidence that several features of fibromyalgia point towards an autoimmune component of the pathogenesis [43]. According to this view, both trauma and infection, which are common triggering events of autoimmunity, can also precede the onset of fibromyalgia [77]. The authors refer to data indicating that various pathogens such as Epstein–Barr virus, herpes simplex virus, known as risk factors for autoimmune diseases, are also involved in the etiology of fibromyalgia [77,78]. It has been reported that in some cases, fibromyalgia can be temporally associated with autoimmune syndrome induced by adjuvants and, similarly to other autoimmune diseases, it has a female predominance [79,80].

The hypothesis is supported by a report describing the association of fibromyalgia with B58, DR5, and DR8 HLA alleles in 55 patients underlining the possibility of genetic susceptibility to the autoimmune responses [81]. HLA association, in particular, the association with HLA class II alleles, could point towards an involvement of CD4^+^ T cell and B cell-mediated antibody responses. Nevertheless, despite some indications pointing in this direction, the evidence of the involvement of autoimmunity among the pathogenic triggering events of fibromyalgia is still limited. [82,83].

The results and hypothesis underlying this view, however, enlighten the possibility that cross- reactive responses, mediated by either B or T cells, can occur upon prolonged chronic pain and systemic inflammation. It is also likely that the development of cross-reactive responses to self-antigens by B and T can preferentially occur in genetically susceptible patients. Moreover, stress-related responses have been postulated to play a role in disease onset and favor the development of autoreactive responses [84,85]. The events linked to autoimmunity could occur as a side manifestation of prolonged pain and inflammation which finally leads to dysregulation of the adaptive immune compartment. However, if autoimmunity is a side component of the disease rather than one of the causative triggering events, what is the link between environmental stress, anxiety and the generation of adaptive immune responses that could finally give rise to autoreactive responses?

An alternative view that links environmental stress events, anxiety, and T cell responses comes from the very novel findings in the field of immunology.

A comprehensive and exhaustive study in a mouse model has been performed, showing that CD4^+^ T cells are essential for the onset and development of stress-induced anxiety behaviors [86]. The authors examined the role of the adaptive immune system in stress-induced behavioral changes by exposing wild-type or immune-deficient mice to electronic foot shock [87,88,89]. The results indicate that adaptive immunity is required for the onset of anxiety-like stress-induced manifestations [90,91,92,93,94,95]. Consistent with previous reports, mice with acute stress exposure had significantly increased frequencies of peripheral CD4^+^ and CD8^+^ T cells compared to non-treated controls [96,97]. Depletion of CD4^+^ or CD8^+^ T cells before exposure to electronic foot shock demonstrated that only depletion of CD4^+^ T cells significantly reversed the stress-induced anxiety-like behavior. Importantly adoptive transfer of CD4^+^ T cells from mice exposed to stress into unexposed recipient mice induced a similar form of anxiety-like behavior. 

RNA sequencing in CD4^+^ T cells from stress-exposed mice showed variation in the expression of genes encoding mitochondrial proteins. The authors concluded that stress exposure induces marked mitochondrial fission in peripheral CD4^+^ T cells associated with abnormal mitochondrial morphology and metabolic dysfunction. Analysis of the effects of critical components of the inflammatory response such as leukotriene B4 (LTB4) and PGE2 showed that LTB4 administration was capable of inducing anxiety-like behavior, which was reversed in part by the removal of CD4^+^ T cells. LTB4 was also capable of inducing mitochondrial fission in CD4^+^ T cells in vitro and could trigger mitochondrial fission in peripheral CD4^+^ T cells in vivo. Mitochondrial fission, in turn, stimulated ex novo xanthine synthesis by CD4^+^ T cells through the induction of transcription factor IRF-1 [98]. IRF-1 accumulation in CD4^+^ T cells is involved in Th1 development and in the maturation of CD8^+^ T-cells. It is also involved in the differentiation and maturation of dendritic cells and in the inhibition of the development of regulatory T (Treg) cells [99,100,101]. On the other hand, patients with immune-mediated diseases such as inflammatory bowel disease showed dysregulated T cell proliferation, which can lead to anxiety-like behavior [102].

This very novel mechanism that remarkably links environmental stress and related mood disorders with CD4^+^ T cell altered functions through mitochondrial fission induced by mediators of inflammation may provide new keys of interpretation for fibromyalgia, which has a debated nature between psychogenic and rheumatic.

It is possible to speculate that the controversial nature of the disease and the association with anxiety and mood disorders, as well as the described association with increased activation of the CD4^+^ compartment, could be linked by a mechanism that involves stress-induced mitochondrial alterations, metabolic dysregulation, and increased Th1 polarization.

## 5. Concluding Remarks

Analysis of literature data provides non-conclusive results on the role of T cells in the development and maintenance of fibromyalgia symptoms. However, it also emerges that in most cases, altered frequency and/or polarization of T cells mainly in the CD4^+^ T cell compartment is observed in patients. The presence of immune-mediated comorbid disease, the detection of autoantibodies and the female prevalence together with data suggesting an association with viral infections have suggested that, in some patients, autoimmune responses occur. However, it is also possible that autoimmunity may represent a side event to the primary alteration of the central pain pathways that finally results in the increase of inflammatory mediators at systemic level. A possible novel key of interpretation may come from recent findings in the field of experimental immunology, providing an intriguing explanation to the link between stress-induced behavioral changes and metabolic and functional alteration in CD4 T cells. These new perspectives may suggest an alternative view that envisages the involvement of variation of the T reactivity beyond the classical antigen-induced activation. This could explain both literature data enlightening alteration of the T cell compartments without providing evidence of T cell responses as main triggering events in fibromyalgia. It may also outline new possible solutions to the controversies about the psychogenic and rheumatic nature of the disease. The data available in the literature and the new perspectives in the field also encourage scientists to apply alternative strategies and advanced technologies for the analysis of the different subsets of T cells. This could shed light on stress-induced morphological changes and metabolic dysfunction that may result in a generalized hyper-activation in the T cell compartment.

## Figures and Tables

**Table 1:** Summary of literature data on T lymphocytes and fibromyalgia.

Cell Subpopulation	Phenotype/Function	Change in FM	Effect of Treatment	Sample Size	References
CD4^+^ T cells	ConA induced IL-2 secretion	reduction	NA	12	Hader et al. (1991) [60]
CD3^+^ T cells	CD69^+^, CD25^+^ activated	reduction	NA	65	Hernanz et al. (1994) [61]
CD3^+^ T cells/NK cells	CD25^+^	Increase	Reduction of CD4^+^HLADR^+^ by low doses IFNα	124 (baseline)	Russel et al. (1999) [62]
CD3^+^ T cells	Proliferation in response to environmental chemicals	Increase	NA	39	Shaklin et al. (2000) [63]
T cells	Number	Increase and association with autoimmune disease posttraumatic stress disorder	NA	2490	Boscarino et al. (2004) [68], Tagoe et al. (2012) [69]
CD8^+^ T cells	-	Reduction	NA	22	Kaufmann et al. (2007) [3]
CD4^+^ T cells	CD4^+^CD25^low^, CD4^+^HLADR^+^	Increase	NA	75	Carvalho et al. (2008) [8]
NKT cells	CD3^+^CD56^+^	Increased in patients with lower depression scores	Modulated by antidepressant54	96	Nugraha et al. (2013) [9]
T cell chemokines	CCl17, CXCL9, CCL22, CXCL11, CCL11	Increased in serum	NA	17	Garcia et al. (2014) [70]
Mucosal Associated invariant T (MAIT)	CD8 MAIT and NKp80, CCR4, CCR7, CXCR1, CD69	Increased	Varied expression by treatment interruption	26	Sugimoto et al. (2015) [72]
T cells	Apoptosis and proinflammatory cytokines secretion	-	Reduction after cannabinoids treatment	Review article	Katchan et al. (2016) [74], Katz et al. (2017) [75]
CD4^+^ T cells	Th1 IFN^+^/TNF^+^	Increased	Reduced by hyperbaric oxygen therapy	36	Guggino et al. (2019) [73]

N/A, not applicable.
